# [Bis(quinolin-2-ylcarbon­yl)amido-κ^3^ *N*,*N*′,*N*′′]bromido­(*N*,*N*-di­methyl­formamide-κ*O*)copper(II)

**DOI:** 10.1107/S1600536814010058

**Published:** 2014-05-10

**Authors:** Md. Serajul Haque Faizi, Pratik Sen

**Affiliations:** a Department of Chemistry, Indian Institute of Technology Kanpur, Kanpur, UP 208 016, India

## Abstract

In the mononuclear title complex, [CuBr(C_20_H_12_N_3_O_2_)(C_3_H_7_NO)], synthesized from the quinoline-derived reduced Schiff base 4-(quinolin-2-ylmeth­yl)amino­phenol, the coordination geometry around Cu^2+^ is distorted square-pyramidal, comprising a bromide anion at the apex [Cu—Br = 2.4671 (5) Å]. The base of the pyramid is built up from one di­methyl­formamide O-atom donor [Cu—O = 2.078 (2) Å] and three N-atom donors from the monoanionic, tridentate bis­(quinolin-2-ylcarbon­yl)di­imide ligand [Cu—N_di­imide_ = 1.941 (3) Å, and Cu—N_quinol­yl_ = 2.060 (3) and 2.049 (3) Å]. An intra­molecular C—H⋯O occurs. In the crystal, weak methyl and aromatic C—H⋯Br and formyl C—H⋯O_carbon­yl_ hydrogen-bonding inter­actions generate an overall layered structure lying parallel to (001).

## Related literature   

For applications of the title complex and related structures, see: Castro *et al.* (1990 ▶, 1991 ▶, 1999 ▶); Vangdal *et al.* (2002 ▶); Sahu *et al.* (2010 ▶); Carlucci *et al.* (2011 ▶); Calatayud *et al.* (2000 ▶); Lebon *et al.* (1998 ▶).
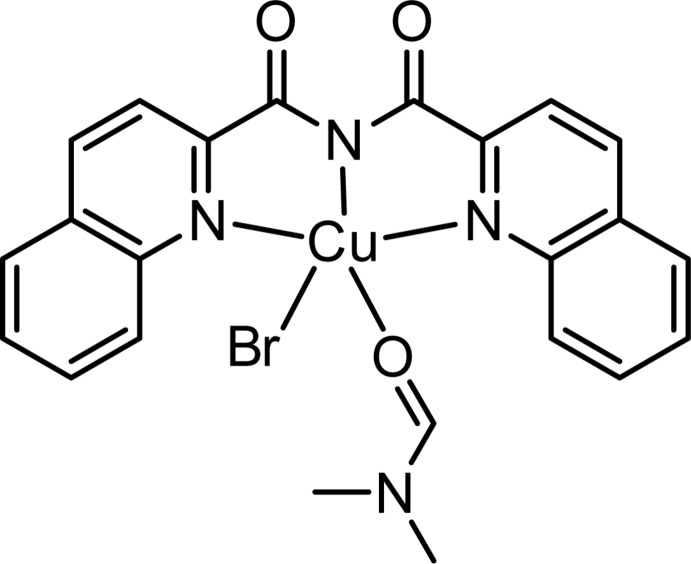


## Experimental   

### 

#### Crystal data  

[CuBr(C_20_H_12_N_3_O_2_)(C_3_H_7_NO)]
*M* *_r_* = 542.87
Monoclinic, 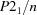

*a* = 9.2137 (6) Å
*b* = 23.5220 (16) Å
*c* = 10.4842 (7) Å
β = 110.284 (1)°
*V* = 2131.3 (2) Å^3^
*Z* = 4
Mo *K*α radiation
μ = 2.93 mm^−1^
*T* = 100 K
0.26 × 0.20 × 0.14 mm

#### Data collection  

Bruker SMART APEX CCD diffractometer
Absorption correction: multi-scan (*SADABS*; Sheldrick, 2004 ▶) *T*_min_ = 0.592, *T*_max_ = 0.681
13799 measured reflections
3753 independent reflections
3223 reflections with *I* > 2σ(*I*)
*R*_int_ = 0.033

#### Refinement  

*R*[*F* ^2^ > 2σ(*F* ^2^)] = 0.034
*wR*(*F* ^2^) = 0.081
*S* = 1.06
3753 reflections
301 parameters
H atoms treated by a mixture of independent and constrained refinement
Δρ_max_ = 0.81 e Å^−3^
Δρ_min_ = −0.45 e Å^−3^

### 

Data collection: *SMART* (Bruker, 2003 ▶); cell refinement: *SAINT* (Bruker, 2003 ▶); data reduction: *SAINT*; program(s) used to solve structure: *SIR97* (Altomare *et al.*, 1999 ▶); program(s) used to refine structure: *SHELXL97* (Sheldrick, 2008 ▶); molecular graphics: *DIAMOND* (Brandenberg & Putz, 2006 ▶); software used to prepare material for publication: *DIAMOND*.

## Supplementary Material

Crystal structure: contains datablock(s) global, I. DOI: 10.1107/S1600536814010058/zs2296sup1.cif

Structure factors: contains datablock(s) I. DOI: 10.1107/S1600536814010058/zs2296Isup2.hkl

CCDC reference: 1000734

Additional supporting information:  crystallographic information; 3D view; checkCIF report

## Figures and Tables

**Table 1:** Hydrogen-bond geometry (Å, °)

*D*—H⋯*A*	*D*—H	H⋯*A*	*D*⋯*A*	*D*—H⋯*A*
C23—H23*A*⋯Br1^i^	0.98 (5)	2.87 (4)	3.663 (5)	138 (3)
C15—H15⋯Br1^ii^	0.93	2.82	3.655 (4)	151
C20—H20⋯O1	0.93	2.42	3.059 (4)	126
C22—H22⋯O3^iii^	0.93	2.33	3.060 (4)	135

Symmetry codes: (i) 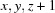; (ii) 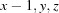; (iii) 
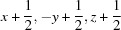
.

**Fig. 1.**
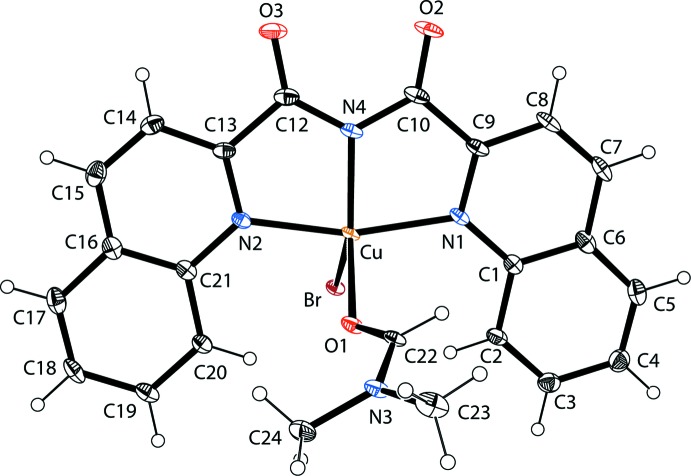
The molecular conformation and atom-numbering scheme for the title complex with non-H atoms drawn as 30% probability displacement ellipsoids.

**Fig. 2.**
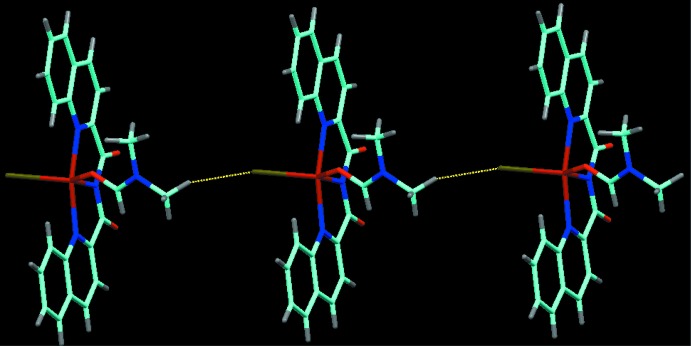
The one-dimensional chain structure in the title complex extending along c, with weak C—H···Br hydrogen bonds shown as dashed lines.

**Fig. 3.**
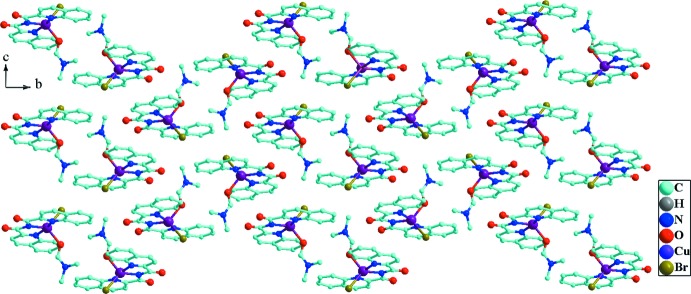
The two-dimensional structure viewed along the c-axial direction.

## supplementary crystallographic information

### 1. Comment

The new ligand bis(2-quinolylcarbonyl)diimide monoanion (BQCD), formed from the quinoliny derived reduced Schiff base 4-(quinolin-2-ylmethyl)aminophenol (R-QMAP), is an important compound widely used in biological applications such as an HIV-1 protease inhibitor and in coordination chemistry (Castro et al., 1990; Castro et al., 1991; Lebon et al., 1998; Castro et al., 1999; Calatayud et al., 2000; Vangdal et al., 2002; Carlucci et al., 2011). In the synthesis of a compound from the reaction of CuBr with BQCD in ethanol with subsequent recrystallization from dimethylformamide generated the title Cu^II^ complex [Cu(C_20_H_12_N_3_O_2_)(C_3_H_7_NO)Br] which contains the monoanionic bis(2-quinolylcarbonyl) diimide ligand (BQCD), one bromido anion and an O-bonded dimethylformamide solvent molecule. The ligand, a bis(2-quinolylcarbonyl)diimide monoanion (BQCD) was formed from a reduced Schiff base 4-(quinolin-2-ylmethyl)aminophenol (^i^R-QMAP), by the breaking of the aminophenol and subsequent oxidation of the –CH_2_– group to a carbonyl group in the presence of dioxygen and copper(I) bromide. This oxidation of the –CH_2_– group to a carbonyl group in the presence of dioxygen and metal salts has previously been reported (Sahu et al., 2010).

In the title mononuclear complex (Fig. 1), the Cu^II^ center is penta-coordinated with a distorted square pyramidal coordination geometry comprising an axial Br anion [Cu—Br = 2.4671 (5) Å] and in the meridional site, a dimethylformamide oxygen atom donor [Cu—O = 2.078 (2) Å] and three N-atom donors from the monoanionic bis(2-quinolylcarbonyl)diimide (BQCD) ligand, viz. two quinolyl nitrogens [Cu—N = 2.060 (3) and 2.049 (3) Å] and one diimide nitrogen [Cu—N = 1.941 (3) Å]. The observed Cu—N bond lengths and bond angles in the title compound are considered normal for this type of Cu^II^ complex, e.g. Cu—N(quinolyl) = 2.035 (5) Å] and [Cu—N(diimide) = 1.966 (5) Å] (Sahu et al., 2010).

In the crystal, a weak intermolecular methyl C23—H···Br1^i^ interaction (Table 1) generates a chain structure extending along the c axial direction (Fig. 2), and is further extended into a two-dimensional sheet structure lying parallel to (001) through aromatic C15—H···Br^ii^ and formyl C22—H···O3^iii^ hydrogen bonds (Fig. 3). Also present in the structure is an intramolecular aromatic C20—H···O1_formyl_ hydrogen bond.

### 2. Experimental

A mixture of reduced Schiff base 4-(quinolin-2-ylmethyl)aminophenol (^i^R-QMAP) (0.10 g, 0.40 mmol), copper(I) bromide (0.060 g, 0.40 mmol), ethanol (5 mL) were stirred vigorously for 30 min, the precipitate was filtered off and dissolved in dimethylformamide and kept for crystallization. Crystals suitable for X-ray analysis were obtained within a week by slow evaporation of the DMF solvent.

### 3. Refinement

The H-atoms of the methyl group involved in the chain formation (C23) were located in a difference-Fourier and were fully refined. All other H-atoms were positioned geometrically and refined using a riding model with C—H = 0.93–0.96 Å and *U*_iso_(H) = 1.2*U*_eq_(aromatic C) or 1.5*U*_eq_(methyl C).

### Crystal data

**Table:**

[CuBr(C_20_H_12_N_3_O_2_)(C_3_H_7_NO)]	*F*(000) = 1092
*M**_r_* = 542.87	*D*_x_ = 1.692 Mg m^−^^3^
Monoclinic, *P*2_1_/*n*	Mo *K*α radiation, λ = 0.71073 Å
Hall symbol: -P 2yn	Cell parameters from 7192 reflections
*a* = 9.2137 (6) Å	θ = 2.2–28.3°
*b* = 23.5220 (16) Å	µ = 2.93 mm^−^^1^
*c* = 10.4842 (7) Å	*T* = 100 K
β = 110.284 (1)°	Needle, red
*V* = 2131.3 (2) Å^3^	0.26 × 0.20 × 0.14 mm
*Z* = 4	

### Data collection

**Table:**

Bruker SMART APEX CCD diffractometer	3753 independent reflections
Radiation source: fine-focus sealed tube	3223 reflections with *I* > 2σ(*I*)
Graphite monochromator	*R*_int_ = 0.033
ω and φ scans	θ_max_ = 25.0°, θ_min_ = 2.2°
Absorption correction: multi-scan (*SADABS*; Sheldrick, 2004)	*h* = −10→10
*T*_min_ = 0.592, *T*_max_ = 0.681	*k* = −27→27
13799 measured reflections	*l* = −12→10

### Refinement

**Table:**

Refinement on *F*^2^	Primary atom site location: structure-invariant direct methods
Least-squares matrix: full	Secondary atom site location: difference Fourier map
*R*[*F*^2^ > 2σ(*F*^2^)] = 0.034	Hydrogen site location: inferred from neighbouring sites
*wR*(*F*^2^) = 0.081	H atoms treated by a mixture of independent and constrained refinement
*S* = 1.06	*w* = 1/[σ^2^(*F*_o_^2^) + (0.0432*P*)^2^ + 2.2471*P*] where *P* = (*F*_o_^2^ + 2*F*_c_^2^)/3
3753 reflections	(Δ/σ)_max_ = 0.001
301 parameters	Δρ_max_ = 0.81 e Å^−^^3^
0 restraints	Δρ_min_ = −0.45 e Å^−^^3^

### Special details

**Table:**

Geometry. All e.s.d.'s (except the e.s.d. in the dihedral angle between two l.s. planes) are estimated using the full covariance matrix. The cell e.s.d.'s are taken into account individually in the estimation of e.s.d.'s in distances, angles and torsion angles; correlations between e.s.d.'s in cell parameters are only used when they are defined by crystal symmetry. An approximate (isotropic) treatment of cell e.s.d.'s is used for estimating e.s.d.'s involving l.s. planes.
Refinement. Refinement of *F*^2^ against ALL reflections. The weighted *R*-factor *wR* and goodness of fit *S* are based on *F*^2^, conventional *R*-factors *R* are based on *F*, with *F* set to zero for negative *F*^2^. The threshold expression of *F*^2^ > σ(*F*^2^) is used only for calculating *R*-factors(gt) *etc*. and is not relevant to the choice of reflections for refinement. *R*-factors based on *F*^2^ are statistically about twice as large as those based on *F*, and *R*- factors based on ALL data will be even larger.

### Fractional atomic coordinates and isotropic or equivalent isotropic displacement parameters (Å^2^)

**Table:**

	*x*	*y*	*z*	*U*_iso_*/*U*_eq_	
C1	1.2311 (4)	0.17727 (13)	0.9003 (3)	0.0186 (7)	
C2	1.2627 (4)	0.11829 (14)	0.9138 (3)	0.0225 (8)	
H2	1.1818	0.0922	0.8872	0.027*	
C3	1.4112 (4)	0.09975 (15)	0.9655 (3)	0.0276 (8)	
H3	1.4309	0.0609	0.9747	0.033*	
C4	1.5355 (4)	0.13798 (16)	1.0051 (4)	0.0305 (9)	
H4	1.6365	0.1244	1.0397	0.037*	
C5	1.5086 (4)	0.19471 (16)	0.9931 (4)	0.0294 (9)	
H5	1.5916	0.2199	1.0191	0.035*	
C6	1.3571 (4)	0.21600 (14)	0.9420 (3)	0.0245 (8)	
C7	1.3227 (5)	0.27458 (15)	0.9297 (4)	0.0298 (9)	
H7	1.4024	0.3011	0.9549	0.036*	
C8	1.1742 (4)	0.29206 (14)	0.8811 (4)	0.0277 (8)	
H8	1.1511	0.3307	0.8747	0.033*	
C9	1.0551 (4)	0.25196 (13)	0.8405 (3)	0.0219 (8)	
C10	0.8890 (4)	0.27133 (14)	0.7873 (3)	0.0238 (8)	
N4	0.7906 (3)	0.22593 (11)	0.7571 (3)	0.0210 (6)	
C12	0.6353 (4)	0.23086 (14)	0.7065 (3)	0.0249 (8)	
C13	0.5577 (4)	0.17340 (14)	0.6804 (3)	0.0207 (7)	
C14	0.3975 (4)	0.17069 (15)	0.6286 (3)	0.0255 (8)	
H14	0.3388	0.2038	0.6070	0.031*	
C15	0.3275 (4)	0.11898 (16)	0.6097 (3)	0.0292 (8)	
H15	0.2202	0.1164	0.5748	0.035*	
C16	0.4180 (4)	0.06968 (15)	0.6433 (3)	0.0235 (8)	
C17	0.3511 (4)	0.01469 (16)	0.6265 (4)	0.0307 (9)	
H17	0.2441	0.0106	0.5903	0.037*	
C18	0.4423 (4)	−0.03185 (15)	0.6630 (4)	0.0304 (9)	
H18	0.3975	−0.0678	0.6510	0.036*	
C19	0.6035 (4)	−0.02662 (14)	0.7185 (3)	0.0269 (8)	
H19	0.6643	−0.0590	0.7454	0.032*	
C20	0.6726 (4)	0.02559 (14)	0.7337 (3)	0.0219 (7)	
H20	0.7799	0.0285	0.7688	0.026*	
C21	0.5808 (4)	0.07501 (14)	0.6959 (3)	0.0195 (7)	
C22	0.9923 (4)	0.10622 (13)	1.0635 (3)	0.0191 (7)	
H22	1.0592	0.1372	1.0867	0.023*	
C23	1.0494 (6)	0.09139 (18)	1.3042 (4)	0.0348 (10)	
C24	0.8692 (5)	0.02789 (15)	1.1333 (4)	0.0341 (9)	
H24A	0.8694	0.0116	1.2173	0.051*	
H24B	0.9036	0.0000	1.0833	0.051*	
H24C	0.7663	0.0399	1.0806	0.051*	
N1	1.0803 (3)	0.19603 (11)	0.8492 (3)	0.0183 (6)	
N2	0.6487 (3)	0.12808 (11)	0.7128 (3)	0.0180 (6)	
N3	0.9720 (3)	0.07624 (11)	1.1617 (3)	0.0219 (6)	
O1	0.9264 (3)	0.09523 (9)	0.9413 (2)	0.0223 (5)	
O2	0.8575 (3)	0.32184 (9)	0.7751 (3)	0.0337 (6)	
O3	0.5551 (3)	0.27363 (10)	0.6817 (3)	0.0399 (7)	
Cu1	0.87800 (5)	0.150012 (15)	0.77633 (4)	0.01711 (12)	
Br1	0.93628 (4)	0.090477 (13)	0.60641 (3)	0.01868 (11)	
H23A	0.972 (5)	0.1009 (16)	1.346 (4)	0.033 (11)*	
H23B	1.114 (5)	0.0610 (18)	1.354 (4)	0.038 (11)*	
H23C	1.111 (4)	0.1234 (17)	1.315 (4)	0.030 (11)*	

### Atomic displacement parameters (Å^2^)

**Table:**

	*U*^11^	*U*^22^	*U*^33^	*U*^12^	*U*^13^	*U*^23^
C1	0.0256 (18)	0.0192 (17)	0.0137 (16)	−0.0035 (14)	0.0102 (14)	−0.0026 (13)
C2	0.032 (2)	0.0153 (17)	0.0189 (17)	−0.0025 (14)	0.0067 (15)	0.0008 (13)
C3	0.033 (2)	0.0251 (19)	0.0238 (18)	0.0015 (16)	0.0085 (16)	0.0017 (15)
C4	0.028 (2)	0.034 (2)	0.0267 (19)	−0.0001 (17)	0.0058 (16)	0.0017 (16)
C5	0.030 (2)	0.034 (2)	0.0247 (19)	−0.0120 (17)	0.0100 (16)	−0.0072 (16)
C6	0.036 (2)	0.0228 (18)	0.0163 (17)	−0.0076 (16)	0.0110 (16)	−0.0064 (14)
C7	0.040 (2)	0.0210 (19)	0.030 (2)	−0.0163 (17)	0.0143 (18)	−0.0087 (15)
C8	0.045 (2)	0.0115 (16)	0.031 (2)	−0.0079 (16)	0.0188 (18)	−0.0034 (14)
C9	0.038 (2)	0.0120 (16)	0.0193 (17)	−0.0020 (14)	0.0138 (16)	−0.0019 (13)
C10	0.042 (2)	0.0134 (17)	0.0223 (18)	0.0008 (15)	0.0191 (17)	−0.0008 (13)
N4	0.0318 (17)	0.0102 (13)	0.0229 (15)	0.0016 (12)	0.0120 (13)	0.0002 (11)
C12	0.033 (2)	0.0202 (18)	0.0254 (19)	0.0062 (16)	0.0154 (16)	0.0062 (14)
C13	0.0279 (19)	0.0181 (17)	0.0183 (16)	0.0049 (15)	0.0107 (15)	0.0050 (13)
C14	0.0264 (19)	0.0274 (19)	0.0229 (18)	0.0069 (16)	0.0090 (15)	0.0060 (15)
C15	0.0232 (19)	0.041 (2)	0.0225 (18)	−0.0011 (17)	0.0068 (16)	0.0034 (16)
C16	0.0286 (19)	0.0279 (19)	0.0141 (16)	−0.0057 (15)	0.0073 (15)	−0.0014 (14)
C17	0.029 (2)	0.037 (2)	0.0237 (18)	−0.0135 (17)	0.0067 (16)	−0.0029 (16)
C18	0.040 (2)	0.0223 (19)	0.0288 (19)	−0.0165 (17)	0.0123 (18)	−0.0041 (15)
C19	0.040 (2)	0.0159 (17)	0.0259 (18)	−0.0040 (15)	0.0120 (17)	−0.0017 (14)
C20	0.0268 (18)	0.0182 (17)	0.0219 (17)	−0.0022 (14)	0.0100 (15)	−0.0020 (13)
C21	0.0280 (19)	0.0188 (17)	0.0133 (16)	−0.0031 (14)	0.0093 (14)	−0.0027 (13)
C22	0.0299 (19)	0.0083 (15)	0.0213 (18)	0.0001 (14)	0.0115 (15)	−0.0014 (13)
C23	0.061 (3)	0.028 (2)	0.0172 (18)	0.000 (2)	0.015 (2)	−0.0017 (16)
C24	0.039 (2)	0.026 (2)	0.036 (2)	−0.0036 (17)	0.0112 (18)	0.0131 (17)
N1	0.0292 (16)	0.0115 (14)	0.0165 (13)	−0.0043 (11)	0.0107 (12)	−0.0022 (10)
N2	0.0246 (15)	0.0150 (14)	0.0163 (14)	−0.0011 (11)	0.0095 (12)	0.0005 (11)
N3	0.0347 (17)	0.0133 (14)	0.0193 (14)	−0.0003 (12)	0.0114 (13)	0.0018 (11)
O1	0.0359 (14)	0.0137 (11)	0.0151 (12)	−0.0038 (10)	0.0061 (11)	0.0021 (9)
O2	0.0499 (17)	0.0094 (13)	0.0490 (17)	0.0040 (11)	0.0262 (14)	0.0010 (11)
O3	0.0388 (16)	0.0192 (14)	0.0627 (19)	0.0107 (12)	0.0188 (14)	0.0121 (13)
Cu1	0.0243 (2)	0.0083 (2)	0.0190 (2)	−0.00018 (15)	0.00791 (17)	0.00038 (15)
Br1	0.02579 (19)	0.01385 (17)	0.01721 (17)	0.00011 (13)	0.00847 (14)	−0.00126 (12)

### Geometric parameters (Å, º)

**Table:**

Br1—Cu1	2.4671 (5)	C13—C14	1.386 (5)
Cu1—O1	2.078 (2)	C14—C15	1.359 (5)
Cu1—N1	2.060 (3)	C15—C16	1.400 (5)
Cu1—N2	2.049 (3)	C16—C17	1.417 (5)
Cu1—N4	1.941 (3)	C16—C21	1.413 (5)
O1—C22	1.240 (4)	C17—C18	1.352 (5)
O2—C10	1.219 (4)	C18—C19	1.400 (5)
O3—C12	1.222 (4)	C19—C20	1.367 (5)
N1—C1	1.377 (5)	C20—C21	1.411 (5)
N1—C9	1.334 (4)	C2—H2	0.9300
N2—C13	1.326 (4)	C3—H3	0.9300
N2—C21	1.380 (4)	C4—H4	0.9300
N3—C22	1.314 (4)	C5—H5	0.9300
N3—C23	1.459 (5)	C7—H7	0.9300
N3—C24	1.444 (5)	C8—H8	0.9300
N4—C10	1.365 (4)	C14—H14	0.9300
N4—C12	1.348 (5)	C15—H15	0.9300
C1—C2	1.415 (5)	C17—H17	0.9300
C1—C6	1.420 (5)	C18—H18	0.9300
C2—C3	1.357 (5)	C19—H19	0.9300
C3—C4	1.401 (5)	C20—H20	0.9300
C4—C5	1.355 (5)	C22—H22	0.9300
C5—C6	1.403 (5)	C23—H23A	0.98 (5)
C6—C7	1.410 (5)	C23—H23B	0.96 (4)
C7—C8	1.348 (6)	C23—H23C	0.93 (4)
C8—C9	1.397 (5)	C24—H24A	0.9600
C9—C10	1.506 (5)	C24—H24B	0.9600
C12—C13	1.509 (5)	C24—H24C	0.9600
			
Br1—Cu1—O1	102.21 (6)	C15—C16—C17	122.0 (3)
Br1—Cu1—N1	99.83 (8)	C15—C16—C21	118.9 (3)
Br1—Cu1—N2	94.61 (8)	C17—C16—C21	119.1 (3)
Br1—Cu1—N4	129.33 (9)	C16—C17—C18	120.2 (4)
O1—Cu1—N1	96.41 (11)	C17—C18—C19	120.8 (3)
O1—Cu1—N2	90.80 (11)	C18—C19—C20	120.8 (3)
O1—Cu1—N4	128.23 (11)	C19—C20—C21	119.9 (3)
N1—Cu1—N2	162.09 (11)	N2—C21—C16	120.2 (3)
N1—Cu1—N4	81.05 (12)	N2—C21—C20	120.6 (3)
N2—Cu1—N4	81.60 (11)	C16—C21—C20	119.2 (3)
Cu1—O1—C22	128.2 (2)	O1—C22—N3	123.1 (3)
Cu1—N1—C1	129.6 (2)	C1—C2—H2	120.00
Cu1—N1—C9	112.3 (2)	C3—C2—H2	120.00
C1—N1—C9	118.1 (3)	C2—C3—H3	119.00
Cu1—N2—C13	111.7 (2)	C4—C3—H3	119.00
Cu1—N2—C21	129.8 (2)	C3—C4—H4	120.00
C13—N2—C21	118.4 (3)	C5—C4—H4	120.00
C22—N3—C23	121.3 (3)	C4—C5—H5	120.00
C22—N3—C24	121.5 (3)	C6—C5—H5	120.00
C23—N3—C24	117.2 (3)	C6—C7—H7	120.00
Cu1—N4—C10	118.5 (2)	C8—C7—H7	120.00
Cu1—N4—C12	117.8 (2)	C7—C8—H8	120.00
C10—N4—C12	123.6 (3)	C9—C8—H8	120.00
N1—C1—C2	119.9 (3)	C13—C14—H14	120.00
N1—C1—C6	121.4 (3)	C15—C14—H14	120.00
C2—C1—C6	118.7 (3)	C14—C15—H15	120.00
C1—C2—C3	119.9 (3)	C16—C15—H15	120.00
C2—C3—C4	121.3 (3)	C16—C17—H17	120.00
C3—C4—C5	120.0 (4)	C18—C17—H17	120.00
C4—C5—C6	120.9 (4)	C17—C18—H18	120.00
C1—C6—C5	119.2 (3)	C19—C18—H18	120.00
C1—C6—C7	117.7 (3)	C18—C19—H19	120.00
C5—C6—C7	123.1 (4)	C20—C19—H19	120.00
C6—C7—C8	120.0 (4)	C19—C20—H20	120.00
C7—C8—C9	119.8 (3)	C21—C20—H20	120.00
N1—C9—C8	123.1 (3)	O1—C22—H22	118.00
N1—C9—C10	117.0 (3)	N3—C22—H22	118.00
C8—C9—C10	119.9 (3)	N3—C23—H23A	110 (2)
O2—C10—N4	128.6 (3)	N3—C23—H23B	111 (2)
O2—C10—C9	120.5 (3)	N3—C23—H23C	113 (2)
N4—C10—C9	110.9 (3)	H23A—C23—H23B	110 (3)
O3—C12—N4	129.5 (3)	H23A—C23—H23C	106 (3)
O3—C12—C13	119.0 (3)	H23B—C23—H23C	108 (4)
N4—C12—C13	111.5 (3)	N3—C24—H24A	109.00
N2—C13—C12	117.1 (3)	N3—C24—H24B	110.00
N2—C13—C14	123.8 (3)	N3—C24—H24C	109.00
C12—C13—C14	119.0 (3)	H24A—C24—H24B	109.00
C13—C14—C15	119.0 (3)	H24A—C24—H24C	109.00
C14—C15—C16	119.6 (3)	H24B—C24—H24C	110.00
			
C6—C1—C2—C3	−0.1 (5)	C12—C13—C14—C15	−177.7 (3)
N1—C1—C2—C3	−179.3 (3)	N2—C13—C14—C15	0.4 (5)
C2—C1—C6—C5	1.0 (5)	C12—C13—N2—C21	177.2 (3)
C2—C1—C6—C7	−179.1 (3)	C12—C13—N2—Cu1	−5.0 (4)
N1—C1—C6—C5	−179.8 (3)	C14—C13—N2—C21	−0.9 (5)
N1—C1—C6—C7	0.1 (5)	C14—C13—N2—Cu1	177.0 (3)
C2—C1—N1—C9	178.7 (3)	C13—C14—C15—C16	0.1 (5)
C2—C1—N1—Cu1	−2.9 (5)	C14—C15—C16—C17	179.6 (3)
C6—C1—N1—C9	−0.4 (5)	C14—C15—C16—C21	−0.1 (5)
C6—C1—N1—Cu1	177.9 (2)	C15—C16—C17—C18	−178.5 (3)
C1—C2—C3—C4	−0.6 (5)	C21—C16—C17—C18	1.2 (5)
C2—C3—C4—C5	0.4 (6)	C15—C16—C21—C20	178.0 (3)
C3—C4—C5—C6	0.5 (6)	C15—C16—C21—N2	−0.4 (5)
C4—C5—C6—C1	−1.3 (5)	C17—C16—C21—C20	−1.6 (5)
C4—C5—C6—C7	178.9 (4)	C17—C16—C21—N2	179.9 (3)
C1—C6—C7—C8	0.8 (5)	C16—C17—C18—C19	0.5 (6)
C5—C6—C7—C8	−179.3 (4)	C17—C18—C19—C20	−1.8 (6)
C6—C7—C8—C9	−1.3 (6)	C18—C19—C20—C21	1.4 (5)
C7—C8—C9—C10	179.5 (3)	C19—C20—C21—C16	0.4 (5)
C7—C8—C9—N1	1.0 (6)	C19—C20—C21—N2	178.8 (3)
C8—C9—C10—N4	−178.3 (3)	C16—C21—N2—C13	0.9 (5)
C8—C9—C10—O2	2.4 (5)	C16—C21—N2—Cu1	−176.5 (2)
N1—C9—C10—N4	0.4 (4)	C20—C21—N2—C13	−177.5 (3)
N1—C9—C10—O2	−179.0 (3)	C20—C21—N2—Cu1	5.1 (5)
C8—C9—N1—C1	−0.1 (5)	O1—C22—N3—C23	−178.7 (3)
C8—C9—N1—Cu1	−178.7 (3)	O1—C22—N3—C24	−0.4 (5)
C10—C9—N1—C1	−178.6 (3)	N3—C22—O1—Cu1	155.7 (2)
C10—C9—N1—Cu1	2.7 (4)	C1—N1—Cu1—N4	178.0 (3)
C9—C10—N4—C12	−179.4 (3)	C1—N1—Cu1—N2	163.4 (3)
C9—C10—N4—Cu1	−3.6 (4)	C1—N1—Cu1—O1	50.2 (3)
O2—C10—N4—C12	−0.0 (6)	C1—N1—Cu1—Br1	−53.4 (3)
O2—C10—N4—Cu1	175.7 (3)	C9—N1—Cu1—N4	−3.6 (2)
C10—N4—C12—C13	178.7 (3)	C9—N1—Cu1—N2	−18.2 (5)
C10—N4—C12—O3	−1.9 (6)	C9—N1—Cu1—O1	−131.4 (2)
Cu1—N4—C12—C13	3.0 (4)	C9—N1—Cu1—Br1	125.0 (2)
Cu1—N4—C12—O3	−177.6 (3)	C13—N2—Cu1—N4	5.1 (2)
C10—N4—Cu1—N1	4.1 (2)	C13—N2—Cu1—N1	19.6 (5)
C10—N4—Cu1—N2	179.6 (3)	C13—N2—Cu1—O1	133.6 (2)
C10—N4—Cu1—O1	95.2 (3)	C13—N2—Cu1—Br1	−124.1 (2)
C10—N4—Cu1—Br1	−91.4 (2)	C21—N2—Cu1—N4	−177.4 (3)
C12—N4—Cu1—N1	−180.0 (3)	C21—N2—Cu1—N1	−162.8 (3)
C12—N4—Cu1—N2	−4.5 (2)	C21—N2—Cu1—O1	−48.8 (3)
C12—N4—Cu1—O1	−88.8 (3)	C21—N2—Cu1—Br1	53.5 (3)
C12—N4—Cu1—Br1	84.6 (3)	C22—O1—Cu1—N4	−45.9 (3)
N4—C12—C13—C14	179.8 (3)	C22—O1—Cu1—N1	37.8 (3)
N4—C12—C13—N2	1.6 (4)	C22—O1—Cu1—N2	−125.8 (3)
O3—C12—C13—C14	0.3 (5)	C22—O1—Cu1—Br1	139.3 (3)
O3—C12—C13—N2	−177.9 (3)		

### Hydrogen-bond geometry (Å, º)

**Table:**

*D*—H···*A*	*D*—H	H···*A*	*D*···*A*	*D*—H···*A*
C23—H23*A*···Br1^i^	0.98 (5)	2.87 (4)	3.663 (5)	138 (3)
C15—H15···Br1^ii^	0.93	2.82	3.655 (4)	151
C20—H20···O1	0.93	2.42	3.059 (4)	126
C22—H22···O3^iii^	0.93	2.33	3.060 (4)	135

Symmetry codes: (i) *x*, *y*, *z*+1; (ii) *x*−1, *y*, *z*; (iii) *x*+1/2, −*y*+1/2, *z*+1/2.
